# Alleviation of Salt Stress in Upland Rice (*Oryza sativa* L. ssp. *indica* cv. Leum Pua) Using Arbuscular Mycorrhizal Fungi Inoculation

**DOI:** 10.3389/fpls.2020.00348

**Published:** 2020-03-26

**Authors:** Rujira Tisarum, Cattarin Theerawitaya, Thapanee Samphumphuang, Kanyamin Polispitak, Panarat Thongpoem, Harminder Pal Singh, Suriyan Cha-um

**Affiliations:** ^1^National Center for Genetic Engineering and Biotechnology (BIOTEC), National Science and Technology Development Agency, Khlong Luang, Thailand; ^2^Devision of Biology, Faculty of Science and Technology, Rajamangala University of Technology Thanyaburi, Khlong Hok, Thailand; ^3^Department of Environment Studies, Faculty of Science, Panjab University, Chandigarh, India

**Keywords:** AMF-inoculation, anthocyanins, cyanidin-3-glucoside, peonidin-3-glucoside, photosynthetic abilities, salt stress, yield attributes

## Abstract

Arbuscular mycorrhizal fungi (AMF) symbionts not only promote the growth of host plant but also alleviate abiotic stresses. This study aimed to investigate the putative role of AMF in salt stress regulation of upland pigmented rice cv. Leum Pua (LP) comparing with Pokkali salt tolerant (positive check). In general, LP is a variety of glutinous rice that contains anthocyanin pigment in the black pericarp, due to which it possesses high antioxidant activities compared to non-pigmented rice. Pot experiment was conducted to evaluate the impact of inoculated AMF, *Glomus etunicatum* (GE), *Glomus geosporum* (GG), and *Glomus mosseae* (GM) strains, in the LP plantlets subjected to 0 (control) or 150 mM NaCl (salt stress) for 2 weeks in comparison with Pokkali (a salt tolerant rice cultivar), which was maintained as a positive check. Root colonization percentage under NaCl conditions ranged from 23 to 30%. Na^+^ content in the flag leaf tissues was increased to 18–35 mg g^–1^ DW after exposure to 150 mM NaCl for 14 days in both inoculated and un-inoculated LP plants, whereas Na:K ratio was very low in cv. Pokkali. Interestingly, sucrose content in the flag leaf tissues of un-inoculated LP plants under salt stress was increased significantly by 50 folds over the control as an indicator of salt stress response, whereas it was unchanged in all AMF treatments. Fructose and free proline in GE inoculated plants under salt stress were accumulated over control by 5.75 and 13.59 folds, respectively, for osmotic adjustment of the cell, thereby maintaining the structure and functions of chlorophyll pigments, F_v_/F_m_, Φ_PSII_, and stomatal function. Shoot height, flag leaf length, number of panicles, panicle length, panicle weight, and 100-grain weight in GE inoculated plants of cv. LP under salt stress were maintained similar to cv. Pokkali. Interestingly, cyanidin-3-glucoside (C3G) and peonidin-3-glucoside (P3G) in the pericarp of cv. LP were regulated by GE inoculation under salt stress conditions. In summary, AMF-inoculation in rice crop is a successful alternative approach to reduce salt toxicity, maintain the yield attributes, and regulate anthocyanins enrichment in the pericarp of grains.

## Introduction

Saline soil affects agricultural productivity in several regions of the world including United States, Argentina, Australia, China, Egypt, India, Iran, Iraq, Pakistan, and Thailand (i.e., an area > 800 million ha; Rengasamy, 2010). It is estimated that 5% or 3.85 million ha of the total cultivated area in the world (77 million ha) is affected by salt stress (Sheng et al., 2008), accounting by nearly 50% of arable land (Wang et al., 2003). By the year 2050, salt affected soil is predicted to be increased up to 16.2 million ha, which may result in food insecurity for world’s population (Yadav et al., 2017). In Southeast Asia, 5.8 million ha arable land has been identified as salt affected (Shrestha, 2006). In Thailand, the problem of saline soil is widely distributed in Northeastern region (1.84 million ha), classifying the agricultural areas as slightly, moderately, and severely salt-affected (Arunin and Pongwichian, 2015).

Arbuscular mycorrhizal fungi (AMF) is one of the symbiotic microorganisms that regulate phosphorus (P) content, growth, and yield of the host plant (Gosling et al., 2006). AMF colonizes with root organs of the host plant, and regulates its photosynthetic abilities, growth characteristics, and abiotic stress tolerance (Panneerselvam et al., 2017; Basu et al., 2018; Mbodj et al., 2018). *Glomus mosseae* (GM), *Glomus geosporum* (GG), *Glomus intraradices*, *Acaulospora* sp., and *Scutellospora* sp. are AMF species that generally colonize with rice (Gosling et al., 2006; Maiti et al., 2013; Zhang et al., 2014; Tisarum et al., 2019). Previous studies have reported a positive relationship between AMF symbiosis and salt defense mechanisms of the host plants (Ruiz-Lozano and Azcón, 2000). For example, ion homeostasis (influx/efflux), compartmentalization (vacuolar storage), and Na^+^ translocation from root to shoot via apoplastic and/or symplastic routes have been regulated by AMF-inoculation (Evelin et al., 2009; Porcel et al., 2012; He and Huang, 2013; Porcel et al., 2016; Yadav et al., 2017; Evelin et al., 2019). Better defense responses in terms of the higher production of free proline, glycine betaine, and soluble sugars in AMF inoculated plants against salt stress have also been reported (Campanelli et al., 2013; Evelin et al., 2013; Garg and Baher, 2013; Talaat and Shawky, 2014). The regulation of proline biosynthesis [pyrroline-5-carboxylate synthetase (P5CS)] and inhibition of proline degradation [proline dehydrogenase (PDH)] are evidently observed when AMF-inoculated plants are exposed to salt stress (Jahromi et al., 2008; Garg and Baher, 2013). Similarly, several antioxidant enzymes, i.e., superoxide dismutase (SOD), catalase (CAT), peroxidase (POD), and ascorbate peroxidase (APX), are upregulated as salt defense responses in AMF inoculated plants under salt stress (Borde et al., 2011; Ruiz-Lozano et al., 2012; Evelin and Kapoor, 2014; Chang et al., 2018).

Rice is an important carbohydrate crop providing a staple food to more than half of the world’s population (Khush, 2005). The crop is highly susceptible to salt stress and its productivity declines even at very low concentrations of salt (Zeng and Shannon, 2000; Grattan et al., 2002). Pokkali cultivar of rice is a salt tolerant cultivar, which is used as a positive check in the screening of salt tolerant rice cultivars (Senadhira et al., 2002) and as a parental line in rice breeding programs conducted to develop salt tolerant traits (de Leon et al., 2016). In Thailand, rice is one of the major cultivating crops, and premium rice varieties with high antioxidant capacities, good cooking quality, and better fragrance are produced and exported globally (Vanavichit et al., 2018). Leum Pua (LP) is one such upland cultivar of glutinous rice with black pericarp, good cooking qualities, fine aroma, excellent flavor, high nutritional values, soft texture, and high antioxidant activities (Kerdphol et al., 2015; Nakaew and Sungthong, 2018; Piyawanitpong et al., 2018; Pornputtapitak et al., 2018; Sansenya et al., 2018; Seekhaw et al., 2018). Upland aerobic rice is known for AMF colonization (Maiti et al., 2011); however, studies investigating salt tolerance ability of AMF colonized upland rice are still lacking. Moreover, the physiological adaptations, i.e., photosynthetic pigments, chlorophyll fluorescence, net photosynthetic rate, stomatal conductance and transpiration rate, morphological responses, and yield attributes, in AMF colonized upland rice under salt stress are critically evaluated as major parameters to investigate salt toxicity (Dodd and Pérez-Alfocea, 2012; Hameed et al., 2014; Latef and Miransari, 2014; Muthukumar et al., 2017; Bhattacharjya et al., 2018). Therefore, the objective of this investigation was to evaluate the potential of three *Glomus* spp. in alleviating the salt stress in pigmented pericarp upland rice (cv. LP) based on physiological and biochemical changes, and yield traits. To the best of our knowledge, this is the first study reporting regulation of salt tolerant abilities in LP using AMF-colonization under salt stress conditions.

## Materials and Methods

### Plant Material, AMF-Inoculation, and Water Deficit Treatment

Seeds of pigmented upland rice cv. “LP (salt sensitive)” and positive check cv. Pokkali (Pok; salt tolerant) were sown in the mixed soil (EC = 2.69 dS m^–1^; pH = 5.5; organic matter = 10.36%; total nitrogen = 0.17%; total phosphorus = 0.07%, and total potassium = 1.19%) for 4 weeks. Healthy seedlings were transplanted into plastic bags containing 2 kg mixed soil in two groups: (a) sterilized soil without AMF and (b) sterilized soil with AMF species: *Glomus etunicatum* (GE; synonym *Claroideoglomus etunicatum*), GG (synonym *Funneliformis geosporum*), and GM (synonym *Funneliformis mosseae*) @ 10 g or 250 spores per plastic bag. Arbuscular mycorrhizal fungus powder was provided by Maejo University, Chiang Mai, Thailand. The powder was inoculated in the soil following the method of Pitaktamrong et al. (2018). The rice plants were grown in a net house under 500–1000 μmol m^–2^ s^–1^ photosynthetic photon flux density (PPFD) with a 10 h d^–1^ photoperiod, 35 ± 2°C (day time)/28 ± 2°C (night time) temperature, and 80 ± 5% RH until booting stage. Thereafter, Pok without AMF (Pok), LP without AMF (LP), and LP with AMF (LP + GE; LP + GG; and LP + GM) were exposed to 0 mM NaCl (control) or 150 mM NaCl (salt stress) for 14 days. Morphological characters, AMF colonization percentage, inorganic ions (Na^+^, K^+^, and Ca^2+^), total phosphorus, osmotic potential, free proline, soluble sugar, chlorophyll content, chlorophyll fluorescence, net photosynthetic rate, stomatal conductance, and transpiration rate were measured in these 10 sets of observations. In addition, the grain yield traits, number of panicles, panicle length, grain fertility percentage, panicle weight, total grain yield per clump, 100-grain weight, and anthocyanin content of cyanidin-3-glucoside (C3G) and peonidin-3-glucoside (P3G) were evaluated in the pericarp of LP rice at the time of harvest.

### AMF Colonization Assay

Fresh roots (3.0 ± 0.5 cm in length) were collected from each set of observations, washed with distilled water, cut into 1.0 cm length and kept in 60% ethanol (used as a storage solution). Roots were washed thrice with distilled water, transferred to 10% KOH, and incubated at 95°C for 30 min. Cleaned roots were again washed with distilled water and stained using 0.05% (*w/v*) Trypan blue for 15 min. AMF-colonization in the roots was observed under light microscope (Zeiss, Germany) to count the arbuscules, vesicles, and mycorrhizal hyphae (Supplementary Figure S1), according to the method of Brundrett et al. (1996).

### Plant Biochemical Analysis

Na^+^, K^+^, and Ca^2+^ were assayed following the modified method of Tanaka et al. (1999) and Hossain et al. (2006). In brief, flag leaf tissues were collected and washed by deionized water to remove surface contaminating ions. The tissue was ground into a powder in liquid nitrogen, extracted with boiling distilled water, and centrifuged at 10,000 × *g* for 10 min. The supernatant was filtered through a 0.45 μm membrane filter (VertiPure^TM^, Vertical^®^). Cellular Na^+^, K^+^, and Ca^2+^ concentrations were determined using Waters HPLC coupled with 432 Conductivity Detector and WATER IC-PACK^TM^ ion-exclusion column (Waters Associates, Millford, MA, United States). Mobile phase, a mixed solution of 0.012 μM nitric acid and 71.73 μM Na-EDTA (ethylene diamine tetraacetic acid disodium salt dehydrate) in deionized water, was used at 0.6 mL min^–1^ flow rate. Na^+^, K^+^, and Ca^2+^ (Sigma, United States) were used as standards.

Available phosphorus (P) was extracted and determined spectrophotometrically as blue molybdate–phosphate complexes under partial reduction with ascorbic acid (Jackson, 1958). Briefly, 100 mg of dried root and flag leaf samples in each treatment were ground, transferred to 1 mL digestion mixture (0.42 g Se, 14 g LiSO_4_⋅2H_2_O added to 350 mL H_2_O_2_, and 420 mL H_2_SO_4_), and then placed on the hot plate (gradually increased from 50 to 150°C) until the mixture turned back. Five-hundred microliters of 72% HClO_4_ was added to each sample and heated until the material became colorless. After cooling, the samples were diluted with equal volume of HClO_4_, filtered (Whatman #42, United Kingdom) and then mixed with 0.5 mL of Barton’s reagent [25 g ammonium molybdate (400 mL), 1.25 g ammonium meta-vanadate (350 mL), and HNO_3_ (250 mL)] for 10 min. Total P (mg g^–1^ DW) was measured at 420 nm by UV-spectrophotometer (HACH DR/4000; Model 48,000, HACH Company, Loveland, CO, United States) using KH_2_PO_4_ as a calibration standard.

Free proline in the flag leaf tissues was extracted and analyzed according to the method of Bates et al. (1973). Fifty milligrams of fresh material was ground with liquid nitrogen in a mortar. The homogenate powder was mixed with 1 mL of aqueous sulfosalicylic acid (3%, *w/v*) and filtered through filter paper (Whatman#1, United Kingdom). The extracted solution was reacted with an equal volume of glacial acetic acid and ninhydrin reagent (1.25 mg ninhydrin in 30 mL glacial acetic acid and 20 mL 6 M H_3_PO_4_) and incubated at 95°C for 1 h. The reaction was terminated by placing the container in an ice bath. The reaction mixture was mixed vigorously with 2 mL of toluene. After cooling to 25°C, the chromophore was measured at 520 nm by UV–Vis spectrophotometer using L-proline as a calibration standard.

Soluble sugars (sucrose, glucose, and fructose) in the flag leaf tissues were assayed following the method of Karkacier et al. (2003). In brief, 50 mg of flag leaf sample was ground in a mortar with liquid nitrogen. One milliliter of nanopure water was added and centrifuged at 10,000 × *g* for 15 min. The supernatant was collected and filtered through a 0.45 μm membrane filter (VertiPure^TM^, Vertical^®^). Twenty microliters of the filtrate was injected into a Waters HPLC equipped with a MetaCarb 87C column and a guard column. Deionized water was used as the mobile phase at a flow rate of 0.5 mL min^–1^. The online detection was performed using a Waters 410 differential refractrometer detector and the data were analyzed by Empower^®^ software. Sucrose, glucose, and fructose (Fluka, United States) were used as the standards.

Total anthocyanins (C3G and P3G) were assayed following the method of Chandra et al. (2001). Hand-dehusked seeds (2 g) were weighed and transferred in capped glass vials and then 1.5 mL of 1% HCl in methanol were added (Supplementary Figure S2). Extracted solution was vortexed and kept in the dark on the shaker (150 r/min) for 12 h in the cold room (8°C). Supernatant was collected and filtered through a 0.45 μm PTFE filter (VertiPure, Vertical Chromatography). Each sample was analyzed by Waters HPLC equipped with a Waters 2998 photodiode array detector set at 520 nm, and fitted with an ODS C_18_ Hypersil column (250 mm × 4.6 mm; 5 μm, Thermo Fisher Scientific Inc., CA, United States). The mobile phase comprised of: Solvent A (0.5% aqueous phosphoric acid, *v/v*), and solvent B (water/acetonitrile/glacial acetic acid/phosphoric acid, 50: 48.5: 1: 0.5, *v/v/v/v*) used as following gradient: 0 min, 20% B (i.e., 80% solvent A and 20% solvent B); 1–26 min, 60% B, 27–30 min, 20% B, 31–35 min, 20% B (80%). Flow rate was set at 0.8 mL min^–1^. Column temperature was set at 30°C and injection volume was 20 μL. C3G and P3G (Sigma–Aldrich, United States) were injected as standards (Supplementary Figure S3).

### Plant Physiological Assay

Osmotic potential in the flag leaf of “LP” rice was measured according to Lanfermeijer et al. (1991). In brief, 100 mg of fresh tissue were chopped into small pieces, transferred to 1.5 mL micro tube, and then crushed using a glass rod. The 20 μL of extracted solution was dropped directly onto a filter paper in an osmometer chamber (5520 Vapro^®^, Wescor, UT, United States) and subsequently, the data were collected. Then, the osmolarity (mmol kg^–1^) was converted to osmotic potential (MPa) using conversion factor of osmotic potential measurement.

Chlorophyll a (Chl_a_), chlorophyll b (Chl_b_), and total chlorophyll (TC) in the flag leaf tissues were analyzed according to the method of Shabala et al. (1998), whereas total carotenoid (C_x+c_) content was assayed following the method of Lichtenthaler (1987). One hundred milligrams of leaf tissue was homogenized in glass vials using 10 mL of 99.5% acetone and blended using a homogenizer (model T25 Ultra Turrax^®^, IKA, Malaysia). The glass vials were sealed with Parafilm^®^ to prevent evaporation, and then stored at 4°C for 48 h. Chl_a_ and Chl_b_ concentrations were measured at 662 and 644 nm, whereas C_x+c_ concentration was measured at 470 nm using UV–Vis spectrophotometer against acetone (99.5%) as a blank.

Chlorophyll fluorescence emission was measured from the adaxial surface of flag leaf using a fluorescence monitoring system (model FMS 2; Hansatech Instruments Ltd., Norfolk, United Kingdom) in the pulse amplitude modulation mode (Loggini et al., 1999). A leaf, kept in dark for 30 min, was initially exposed to the modulated measuring beam of far-red light (LED source) with typical peak at wavelength 735 nm. Original (F_0_) and maximum (F_m_) fluorescence yields were measured under weak modulated red light (<85 μmol m^–2^ s^–1^) with 1.6 s pulses of saturating light (>1500 μmol m^–2^ s^–1^ PPFD) and calculated using FMS software for Windows^®^. The variable fluorescence yield (F_v_) was calculated using the equation: F_v_ = F_m_–F_0_. The ratio of variable to maximum fluorescence (F_v_/F_m_) was calculated as the maximum quantum yield of PSII photochemistry. The photon yield of PSII (Φ_PSII_) in the light was calculated as: F_PSII_ = (F_m_′-F)/F_m_′ after 45 s of illumination, when steady state was achieved (Maxwell and Johnson, 2000).

Net photosynthetic rate (P_n_; μmol m^–2^ s^–1^), transpiration rate (E; mmol H_2_O m^–2^ s^–1^), and stomatal conductance (g_s_; mmol m^–2^ s^–1^) were measured using a Portable Photosynthesis System fitted with an Infra-red Gas Analyzer (IRGA, Model LI 6400, LI-COR^®^ Inc., Lincoln, NE, United States). All parameters were measured continuously by monitoring the content of the air entering and exiting in the IRGA headspace chamber, according to Cha-um et al. (2007).

### Plant Morphological Characterization and Yield Traits

Shoot height, number of leaves, leaf length, leaf width, and number of tillers were measured in LP rice at booting stage (Supplementary Figure S4). Total grain yield, number of panicles, panicle dry weight, panicle length, seed fertility, and 100-grain weight were also evaluated at harvesting stage.

### Statistical Analysis

The experiment was arranged as Completely Randomized Design (CRD) with six biological replicates (*n* = 6) in each treatment. The mean values obtained from 10 set of observations were compared using Tukey’s HSD and analyzed by SPSS software (version 11.5 for Window^®^).

## Results and Discussion

### AMF Colonization and Total P Assay

**FIGURE 1:**
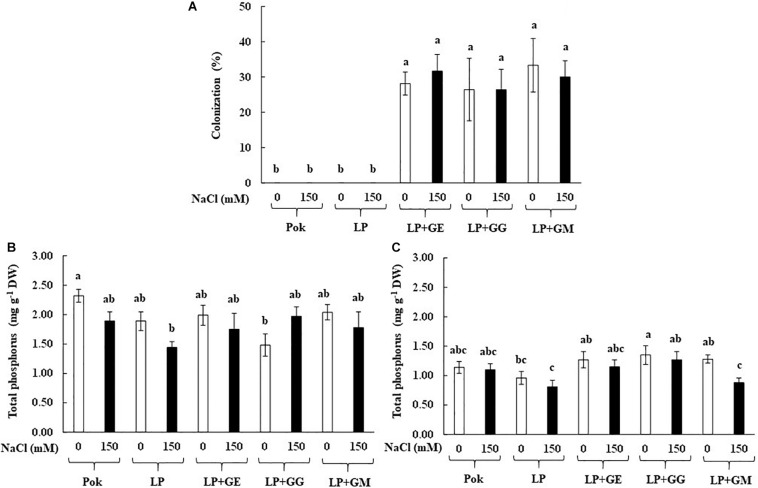
Root AMF colonization **(A)**, total phosphorus in roots **(B)**, and flag leaf tissues **(C)** of “Leum Pua” (LP) plants inoculated with AMF (GE; *Glomus etunicatum*, GG; *G. geosporum*, and GM; *G. mosseae*) at booting stage, and subsequently exposed to salt stress conditions for 14 days. Error bar in each treatment represents by ± SE (*n* = 6). Different letters in each bar represent significant difference at *p* ≤ 0.01 according to Tukey’s HSD.

Arbuscular mycorrhizal fungi colonization percentage in the root tissues of rice cv. LP inoculated with GE, GG, and GM was found to be >26%, irrespective of the salt treatment (Figure 1A). Total P content in the root tissues was greater than that of leaf tissues. Under salt stress, total P content in the root tissues was nearly same in the inoculated and un-inoculated plants. In addition, P content in the root tissues of rice cv. Pokkali (Pok) under control was greater than cv. LP as well as LP + GG (Figure 1B). On the other hand, P content in leaf tissues of LP + GG (1.35 mg g^–1^ DW) was greater than LP (0.81 mg g^–1^ DW) by 1.67 folds (Figure 1C). Moreover, P content in the leaf tissues of LP + GM under salt stress declined by 31.25% over the control.

In the present study, root colonization of AMF inoculated plants was evidently demonstrated in both control and salt stress conditions, whereas it was undetected in un-inoculated plants. As per a previous report inoculation of AM fungus isolated from salt affected soil (Cabo de Gata Natural Park, Spain) showed a positive relation between degree of salt treatments (75 and 150 mM NaCl) and root colonization, and also increased the total P in both shoots and roots (Porcel et al., 2016). Interestingly, root colonization of Rhizophagus *intraradices* (collected), *C. etunicatum*, and *Septoglomus constrictum* was alleviated by salt-treated (66 and 100 mM NaCl) maize plants (Estrada et al., 2013a). In contrast, when *Glomus* spp. collected from rhizosphere of maize plants was inoculated in wheat plants, and subsequently exposed to salt stress, a decline in AM-fungal colonization, especially at high salinity levels (4.7 and 9.4 dS m^–1^) was observed (Talaat and Shawky, 2014). Similarly, in alfalfa, colonization percentage of AMF (*Glomus viscosum*) was sharply declined, in relation to the degree of salt treatments (100–150 mM NaCl) (Campanelli et al., 2013). Colonization percentage of *R. intraradices*, *Massilia* sp. RK4, and their mixtures (collected from rhizosphere of the *Phragmites* sp., Saemangeum reclamation land, South Korea) in maize plants was significantly dropped when subjected to 40 and 80 mM NaCl for 22 days (Krishnamoorthy et al., 2016). In *Leymus chinensis* seedlings, AM root colonization was only detected in AMF inoculation under salt stress (100–200 mM NaCl), whereas it was undetected in un-inoculated plants (Lin et al., 2017).

### Na^+^, K^+^, Ca^2+^, and Na: K Ratio

**FIGURE 2:**
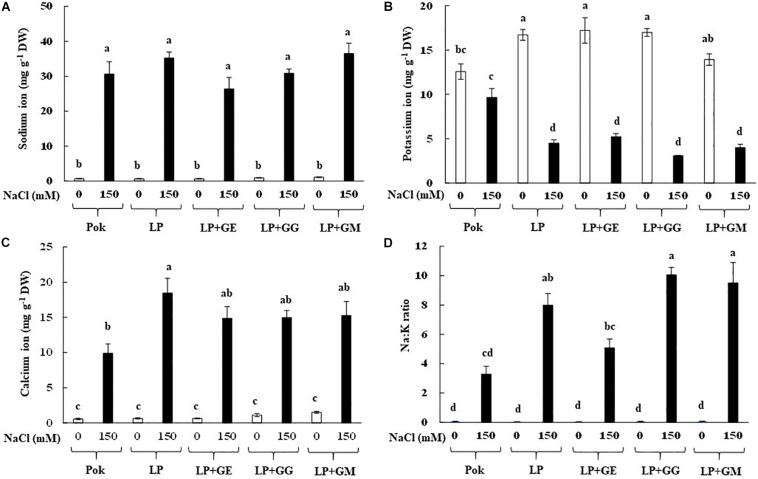
Sodium **(A)**, potassium **(B)**, calcium **(C)** ions, and Na:K ratio **(D)** in flag leaf tissues of “Leum Pua” (LP) plants inoculated with AMF (GE; *Glomus etunicatum*, GG; *G. geosporum*, and GM; *G. mosseae*) at booting stage, and subsequently exposed to salt stress conditions for 14 days. Error bar in each treatment represents by ± SE (*n* = 6). Different letters in each bar represent significant difference at *p* ≤ 0.01 according to Tukey’s HSD.

Na^+^ levels in the flag leaf tissues of rice cvs. Pok (30.66 mg g^–1^ DW) and LP (35.20 mg g^–1^ DW) were increased in response to 150 mM NaCl treatment over the control by 43.20 and 52.50 folds, respectively (Figure 2A). Compared to control, 32.90–40.60 folds increase in Na^+^ level in AMF inoculated plants (GE, GG, and GM) in LP under salt stress was observed. Interestingly, K^+^ in LP was significantly decreased when plants, both with and without AMF, were exposed to 150 mM NaCl salt stress for 14 days, while it was maintained in cv. Pok (Figure 2B). Ca^2+^ was found to be 18.47 mg g^–1^ DW in LP without AMF under salt stress (28.4 folds over control), whereas it was 9.89 mg g^–1^ DW in Pok under salt stress (17.7 folds over control) (Figure 2C). Na:K ratio in salt stressed LP plants, both with and without AMF, was significantly increased; however, it was maintained in cv. Pok (Figure 2D).

Na^+^ and Ca^2+^ were accumulated (by > 10 folds) in the flag leaf tissues of rice cvs. Pok and LP under 150 mM NaCl for 14 days irrespective of AMF-inoculation, whereas K^+^ in LP declined, leading to greater Na^+^:K^+^ ratio. Therefore, Na^+^ enrichment in LP with GE, GG, or GM was lower than that of LP without AMF inoculation. Na^+^ in AMF (*R. intraradices*, *Massilia* sp. RK4, and their mixtures) inoculated maize (cv. Shrunken-2) plants grown under 40 mM NaCl for 17 days was lower than that in the plants without AMF-inoculation (Krishnamoorthy et al., 2016). Similarly, Na^+^ in shoots of AMF (*R. intraradices*, *C. etunicatum*, and *S. constrictum*) inoculated maize plants under 66 and 100 mM NaCl was lower than that in the plants without AMF (Estrada et al., 2013a). In wheat cv. Henta, Na^+^ in shoots of AMF (GM and *Glomus deserticola*) inoculated plants was significantly lesser than in un-inoculated plants and *Gigaspora gergaria* inoculated plants (Abdel-Fattah and Asrar, 2012). In alfalfa cv. icon, low levels of Na^+^ in AMF inoculated plants were demonstrated when compared with the control (Campanelli et al., 2013). In citrus (red tangerine) seedlings, Na^+^ enrichment is generally antagonist with K^+^ when subjected to 100 mM NaCl for 60 days and also, Na^+^ in AMF inoculated plants (GM and *Paraglomus occultum*) was significantly lower than in un-inoculated plants (Wu et al., 2010). Moreover, Na^+^ in AMF inoculated wheat cv. Sids 1 was unchanged, whereas it was increased in cv. Giza 168 over AMF un-inoculated plants, in response to the degree of salinity levels (Talaat and Shawky, 2014). Interestingly, Na^+^ in the shoots of rice cv. Puntal with AMF-inoculation (*C. etunicatum*) was similar to that of the un-inoculated plants, whereas Na^+^ levels in the root tissues of AMF-inoculated plants were higher than control and this involved upregulation of plasma membrane Na^+^/H^+^ antiporter (*OsSOS1*) and high affinity potassium transporter (*OsHKT2;1*) (Porcel et al., 2016). In addition, it was confirmed that expression of vacuolar Na^+^/H^+^ antiporter gene (*LeNHX1*) in the root tissues of salt-stressed tomato was upregulated by AMF (GM) inoculation (He and Huang, 2013).

### Soluble Sugar, Free Proline, Osmotic Potential, and Their Relationship

**FIGURE 3:**
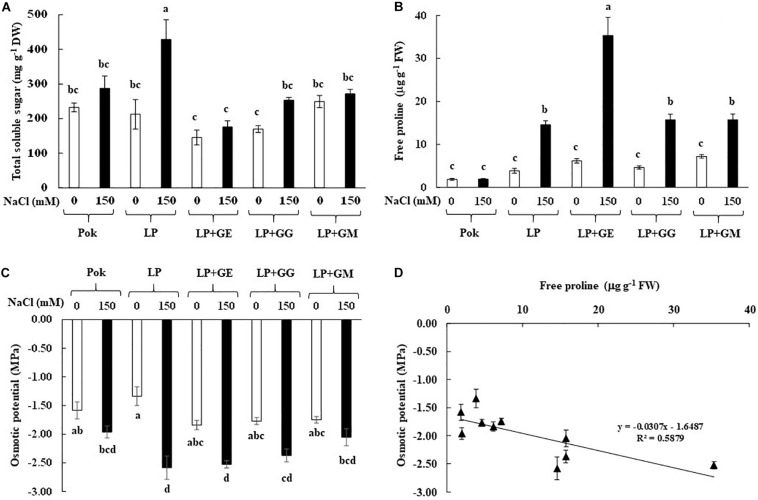
Total soluble sugar **(A)**, free proline **(B)**, osmotic potential in flag leaf **(C)**, and relationship between free proline and osmotic potential in flag leaf **(D)** of “Leum Pua” (LP) plants inoculated with AMF (GE; *Glomus etunicatum*, GG; *G. geosporum*, and GM; *G. mosseae*) at booting stage, and subsequently exposed to salt stress conditions for 14 days. Error bar in each treatment represents by ± SE (*n* = 6). Different letters in each bar represent significant difference at *p* ≤ 0.01 according to Tukey’s HSD.

Sucrose, glucose, and fructose contents in flag leaf tissues were increased when subjected to 150 mM NaCl. Sucrose, glucose and fructose contents in LP plants without AMF under salt stress were peaked at 147.2, 115.9, and 166.7 mg g^–1^ DW and enriched by 62.17, 1.19, and 1.48 folds over the control, respectively (Table 1). Interestingly, fructose in GE-pretreated plants and glucose in GG-pretreated plants of cv. LP exposed to 150 mM NaCl were increased by 13.59 and 1.86 folds over control, respectively (Table 1). Total soluble sugar in LP without AMF was found to be the maximum (2.02 folds over control) when exposed to salt stress. It was maintained at low levels in AMF-pretreated plants similar to that of salt tolerant rice, Pok (Figure 3A). Free proline in Pok was observed to be similar in both control and salt stressed plants. In contrast, it was significantly high in salt stressed plants of cv. LP by 3.79 folds, LP + GM by 2.19 folds, LP + GG by 3.39 folds, and LP + GE by 5.74 folds over control (Figure 3B). Osmotic potential in salt stressed flag leaf of cv. Pok was maintained, whereas it was significantly declined in LP (1.93 folds over control) and LP + GE (1.37 folds over control). Interestingly, it was retained in LP + GG and LP + GM under 150 mM NaCl (Figure 3C). Moreover, a negative relationship between free proline content and osmotic potential was demonstrated (*R*^2^ = 0.5879; Figure 3D).

**TABLE 1:** Sucrose, glucose and fructose contents in “Leum Pua” (LP) plants inoculated with AMF (GE; *Glomus etunicatum*, GG; *G. geosporum*, and GM; *G. mosseae*) of rice cv. at booting stage, and subsequently exposed to salt stress conditions for 14 days.

Treatment	NaCl (mM)	Sucrose (mg g^–1^ DW)	Glucose (mg g^–1^ DW)	Fructose (mg g^–1^ DW)
Pok	0	4.67b	88.03b	139.70ab
	150	23.21b	104.97ab	159.16ab
LP	0	2.36b	97.77b	112.32bc
	150	146.72a	115.91a	166.78a
LP + GE	0	14.81b	53.80c	6.72d
	150	26.52b	58.31c	91.34bc
LP + GG	0	44.94b	47.29c	77.46c
	150	52.31b	88.02b	113.11bc
LP + GM	0	50.27b	77.76bc	121.53b
	150	55.25b	96.13b	120.41b
Significant level		**	**	**

Pok: Pokkali, salt tolerant genotype, positive check. ** represents highly significant difference at *p* ≤ 0.01. Different letters in each column show significant difference at *p* ≤ 0.01 according to Tukey’s HSD.

In rice crop, flag leaf at booting stage is reported to be very sensitive to salt stress (Wankhade et al., 2013). In general, total soluble carbohydrates in the leaf tissues of AMF-colonized plants of trifoliate orange (Zou and Wu, 2011) and chickpea (Garg and Bharti, 2018) were upregulated. In trifoliate orange (*Poncirus trifoliata*), only sucrose was accumulated in the leaves of plants grown under 100 mM NaCl for 7 weeks, whereas glucose and fructose were unchanged even in the plants inoculated with GM and *Glomus versiforme* (Zou and Wu, 2011). In contrast, sucrose in chickpea cvs. PBG 5 (salt tolerant) and BG 256 (salt sensitive), inoculated with *R. intraradices*, was declined in response to the degree of salt stress. Glucose and total soluble sugar in salt tolerant PBG 5 (both with or without AMF-inoculation) were gradually increased when subjected to salt stress (Garg and Bharti, 2018). Total soluble sugars in several plants, i.e., wheat, fenugreek, and two legumes (soybean and cluster bean) grown under salt stress were found to vary in accordance to the degree of salt stress, AMF species, and the symbiotic interactions (Evelin et al., 2013; Datta and Kulkarni, 2014a; Talaat and Shawky, 2014). Interestingly, free proline content in salt tolerant cv. Pok was maintained at low levels, whereas it was enriched by 5.74-folds in salt stressed LP with GE inoculation, over the control. An increasing rate of free proline accumulation in the salt stressed plants has been reported in wheat genotypes, tomato cultivars, and mustard plants in relation to salt-tolerant abilities (Hajiboland et al., 2010; Talaat and Shawky, 2014; Sarwat et al., 2016). On the other hand, free proline enrichment varies according to different species of AMF as seen from the differences observed between GM, *Glomus fasciculatum* (GF), and mixed GM + GF inoculation in *Acacia arabica* (Datta and Kulkarni, 2014b); *R. intraradices*, *C. etunicatum*, and *Septoglomus conicatum* inoculation in maize (Estrada et al., 2013a); and *GM*, *G. deserticola*, and *G. gergaria* inoculation in wheat (Abdel-Fattah and Asrar, 2012). It was confirmed that the P5CS plays a major role in proline biosynthesis under salt stress in both salt tolerant PBG-5 and salt sensitive CSG-9505 genotypes of chickpea (Jahromi et al., 2008; Garg and Baher, 2013). Free proline and total soluble sugars are the major osmolytes in AMF-inoculated plants under salt stress that control the osmotic potential at the cellular level, leading to enhanced salt tolerant ability (Campanelli et al., 2013; Yang et al., 2014; Evelin et al., 2019). Free proline enrichment in the salt stressed plants with AMF inoculation plays a key role as osmotic adjustment (Chun et al., 2018), which confirmed the function as osmolytes by mitigation of NaCl stress in mustard plant (Sarwat et al., 2016).

### Physiological Responses to Salt Stress

**FIGURE 4:**
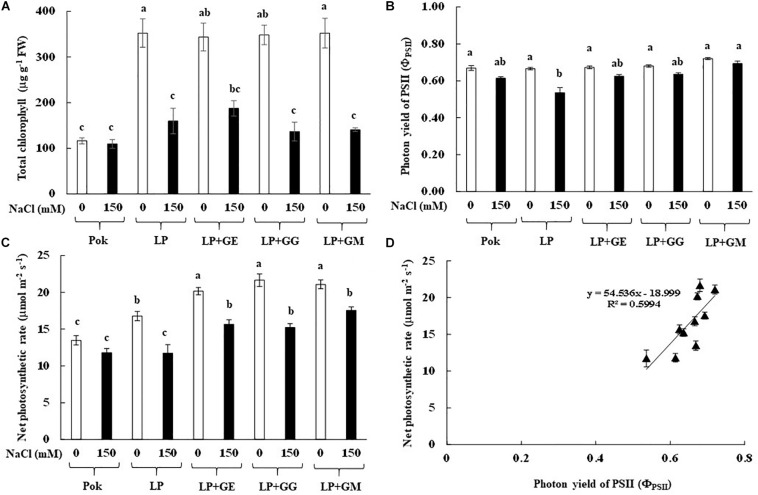
Total chlorophyll content **(A)**, photon yield of PSII **(B)**, net photosynthetic rate **(C)**, and relationship between photon yield of PSII and net photosynthetic rate in flag leaf **(D)** of “Leum Pua” (LP) plants inoculated with AMF (GE; *Glomus etunicatum*, GG; *G. geosporum*, and GM; *G. mosseae*) at booting stage, and subsequently exposed to salt stress conditions for 14 days. Error bar in each treatment represents by ± SE (*n* = 6). Different letters in each bar represent significant difference at *p* ≤ 0.01 according to Tukey’s HSD.

Chl_a_, Chl_b_, and C_x+c_ degradation in cv. Pok under salt stress was low as compared to the cv. LP, where these declined by 64.49, 35.39, and 44.05% over the control, respectively (Table 2). In LP + GE, Chl_a_, Chl_b_, and C_x+c_ in flag leaf tissues were maintained when subjected to salt stress. In contrast, those parameters in LP + GG and LP + GM under salt stress were sharply dropped by ≥50% (Table 2). In addition, TC content in salt stressed plants of cv. Pok was maintained, whereas it was significantly degraded in LP (54.68% over control), LP + GE (45.39% over control), LP + GG (60.79% over control), and LP + GM (60.04% over control) (Figure 4A). F_v_/F_m_, Φ_PSII_, g_s_, and E in the flag leaf of cv. Pok under salt stress were retained, while these were lowered in cv. LP by 25.03, 19.52, 31.03, and 28.89%, respectively (Table 3 and Figure 4B). However, these parameters were maintained by GE, GG, and GM inoculation even when exposed to salt stress (Table 3). P_n_ is a very sensitive parameter to salt stress; however, it was maintained in cv. Pok even under salt stress. In AMF inoculated plants, it was significantly declined by 30.10, 22.31, 29.64, and 16.75% over the control in cv. LP, LP + GE, LP + GG, and LP + GM, respectively (Figure 4C). A positive relation between Φ_PSII_ and P_n_ was also established (*R*^2^ = 0.5994; Figure 4D).

**TABLE 2:** Chlorophyll a (Chl_a_), chlorophyll b (Chl_b_), and total carotenoids (C_x+c_) contents in “Leum Pua” (LP) plants inoculated with AMF (GE; *Glomus etunicatum*, GG; *G. geosporum*, and GM; *G. mosseae*) of rice cv. at booting stage, and subsequently exposed to salt stress conditions for 14 days.

Treatment	NaCl (mM)	Chlorophyll a (μg g^–1^ FW)	Chlorophyll b (μg g^–1^ FW)	Total carotenoids (μg g^–1^ FW)
Pok	0	63.49c	52.88c	7.46bc
	150	58.75c	51.26c	7.05bc
LP	0	233.70a	118.72a	12.60ab
	150	82.99c	76.71bc	6.24c
LP + GE	0	209.94ab	133.76a	16.66a
	150	98.88bc	88.79abc	13.39ab
LP + GG	0	214.61ab	133.82a	16.89a
	150	70.35c	66.22c	6.73c
LP + GM	0	217.01ab	135.36a	19.00a
	150	72.69c	68.10c	7.91*b*c
Significant level		**	**	**

Pok: Pokkali, salt tolerant genotype, positive check. ** represents highly significant difference at *p* ≤ 0.01. Different letters in each column show significant difference at *p* ≤ 0.01 according to Tukey’s HSD.

**TABLE 3:** Maximum quantum yield of PSII (F_v_/F_m_), stomatal conductance (g_s_), and transpiration rate (E) in AMF-inoculated plants (GE; *Glomus etunicatum*, GG; *G. geosporum*, and GM; *G. mosseae*) of rice cv. “Leum Pua” (LP) at booting stage subsequently exposed to salt stress conditions for 14 days.

Treatment	NaCl (mM)	F_v_/F_m_	g_s_ (mmol H_2_O m^–2^ s^–1^)	E (mmol H_2_O m^–2^ s^–1^)
Pok	0	0.865a	0.42a	4.28ab
	150	0.793ab	0.32ab	3.60b
LP	0	0.839ab	0.29b	3.98ab
	150	0.629c	0.20c	2.83c
LP + GE	0	0.858a	0.40ab	4.98a
	150	0.818ab	0.32ab	4.13ab
LP + GG	0	0.842ab	0.39ab	4.77a
	150	0.812ab	0.32ab	4.09ab
LP + GM	0	0.844ab	0.35ab	5.01a
	150	0.818ab	0.31ab	4.20ab
Significant level		**	**	**

Pok: Pokkali, salt tolerant genotype, positive check. ** represents highly significant difference at *p* ≤ 0.01. Different letters in each column show significant difference at *p* ≤ 0.01 according to Tukey’s HSD.

In the present study, chlorophyll pigments: Chl_a_, Chl_b_, and C_x+c_, in GE-inoculated LP plants under salt stress were unchanged, leading to stabilized F_v_/F_m_, Φ_PSII_, and P_n_, whereas these were degraded by >50% over the control in LP without AMF inoculation. Previously, Chl_a_ and Chl_b_ in rice crop cv. Puntal with AMF-inoculation (*C. etunicatum*, isolate EEZ 163) were elevated when compared with non-AMF inoculated crop, both subjected to 150 mM NaCl for 4 weeks (Porcel et al., 2015). Chl_a_ and Chl_b_ in AMF-inoculated plants of false wheatgrass (*L. chinensis* symbiont with GM) and wheat (*Triticum aestivum* L. cvs. Sids 1 and Giza 168 symbiont with a mixture of *Glomus* spp.), were alleviated under both normal and salt stressed conditions (Talaat and Shawky, 2014; Lin et al., 2017). Moreover, plant–microbe interactions are another factor that regulates the salt tolerant abilities in the host plants. For example, Chl_a_, Chl_b_, and C_x+c_ in AMF-inoculated wheat grown with GM under saline soil (860 mg kg^–1^ Na^+^) for 8 and 12 weeks were observed to be higher than those in un-inoculated plants and AMF-inoculated plants with *G. deserticola* and *G. gergaria* (Abdel-Fattah and Asrar, 2012). Consequently, F_v_/F_m_, Φ_PSII_, P_n_, g_s_, and E in AMF-inoculated rice cv. Puntal were promoted under both control and salt stressed conditions (Porcel et al., 2015). In maize, F_v_/F_m_ and g_s_ in plants inoculated with *C. etunicatum*, *R. intraradices*, and *Septoglomus claroideum* under 100 mM NaCl for 30 days were alleviated compared to the un-inoculated plants (Estrada et al., 2013b). In rice crop cv. Puntal, efficiency of PSII and g_s_ in salt stressed plants (75 and 150 mM NaCl for 4 weeks) were significantly improved using *C. etunicatum* isolate EEZ 163 (Porcel et al., 2016). Based on this evidence, it can be suggested that the regulation of osmolytes and antioxidant activities in AMF-inoculated plant grown under salt stress plays a major role in salt defense mechanisms and reduction of electrolyte leakage at the cellular level (Estrada et al., 2013b). Moreover, the photosynthetic efficiencies in AMF-inoculated plants under salt stress are found to be dependent on type of plant species, genotypic variations, AMF genus/species/strain, degree of salt stress, and their interactions (Wu et al., 2010). A positive relationship between Φ_PSII_ and P_n_ with a high correlation coefficient has been observed in rice crop (*R*^2^ = 0.691; Porcel et al., 2015) and black locust (*R*^2^ = 0.789; Zhu et al., 2014), leading to retain the yield attributes.

### Morphological Changes

**FIGURE 5:**
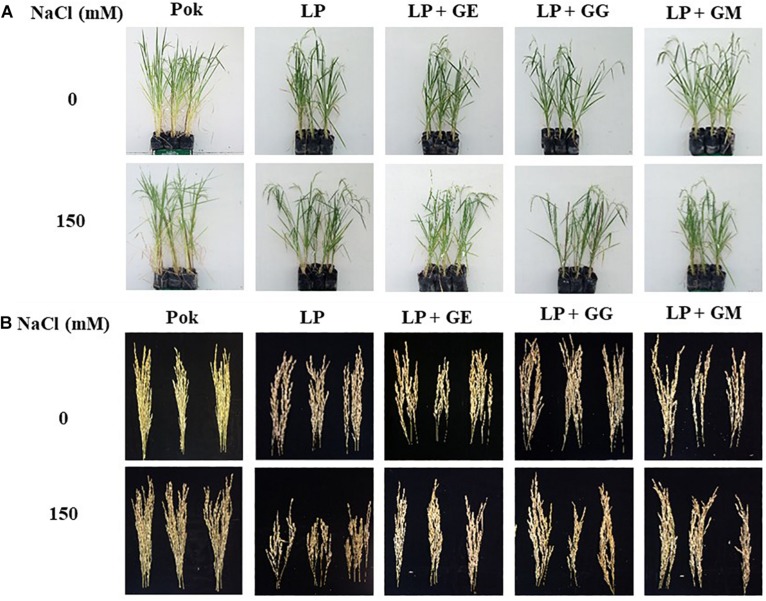
Overall growth performances **(A)** and panicles **(B)** of “Leum Pua” (LP) plants inoculated with AMF (GE; *Glomus etunicatum*, GG; *G. geosporum*, and GM; *G. mosseae*) at booting stage, and subsequently recovered until grain harvesting process.

**FIGURE 6:**
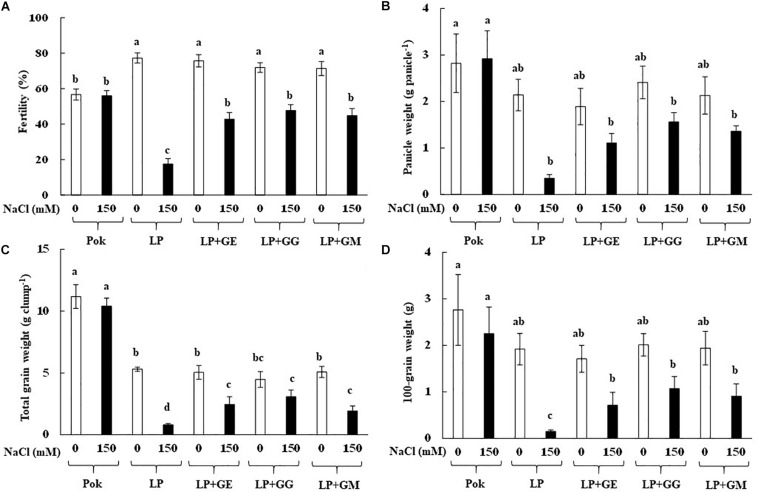
Grain fertility **(A)**, panicle weight **(B)**, total grain weight **(C)**, and 100 grain weight **(D)** of “Leum Pua” (LP) plants inoculated with AMF (GE; *Glomus etunicatum*, GG; *G. geosporum*, and GM; *G. mosseae*) at booting stage, and subsequently recovered until grain harvesting process. Error bar in each treatment represents by ± SE (*n* = 6). Different letters in each bar represent significant difference at *p* ≤ 0.01 according to Tukey’s HSD.

Morphological and phenological characters in cvs. Pok and LP under control and salt stress were also observed (Figure 5). Shoot height, flag leaf length, number of panicle, and panicle length were greater in cv. Pok than in cv. LP. Moreover, these parameters were unchanged when plants were subjected to 150 mM NaCl for 14 days (Table 4). Fertility percentage in cv. Pok under salt stress was unchanged, whereas it was sharply declined by 77.39, 43.48, 33.68, and 37.31% over control in LP, LP + GE, LP + GG, and LP + GM, respectively (Figure 5A). Panicle weight, total grain weight, and 100-grain weight were unchanged in salt stressed rice cv. Pok and LP + GG (Figures 6B–D). In contrast, panicle weight, total grain weight, and 100-grain weight in salt stressed rice cv. LP were significantly declined by 83.65, 84.91, and 92.19%, respectively, over the control. It was confirmed that LP is a salt susceptible variety of rice crop. However, yield attributes such as fertility, panicle weight, total grain weight, and 100-grain weight in AMF-inoculated plants of LP salt stressed rice showed significant improvement compared with un-inoculated plants (Figure 6).

**TABLE 4:** Shoot height, flag leaf length, number of panicles, and panicle length in “Leum Pua” (LP) plants inoculated with AMF (GE; *Glomus etunicatum*, GG; *G. geosporum*, and GM; *G. mosseae*) of rice cv. at booting stage, and subsequently exposed to salt stress conditions for 14 days.

Treatment	NaCl (mM)	Shoot height (cm)	Flag leaf length (cm)	Number of panicles	Panicle length (cm)
Pok	0	126.3a	49.5a	5.3a	27.1a
	150	115.3a	48.7a	4.2ab	26.8a
LP	0	87.4b	29.7b	3.5b	22.6ab
	150	82.1b	26.8b	3.0b	18.8b
LP + GE	0	86.3b	22.0b	3.0b	23.8ab
	150	82.3b	20.8b	3.0b	21.1ab
LP + GG	0	88.2b	27.9b	3.0b	22.4ab
	150	84.0b	24.0b	2.3b	21.4ab
LP + GM	0	88.2b	28.3b	3.2b	24.8a
	150	87.3b	26.7b	2.5b	21.7ab
Significant level		**	**	**	**

Pok: Pokkali, salt tolerant genotype, positive check. ** represents highly significant difference at *p* ≤ 0.01. Different letters in each column show significant difference at *p* ≤ 0.01 according to Tukey’s HSD.

In the present study, shoot height in LP plants exposed to salt stress was unchanged irrespective of the AMF inoculation. In general, shoot height improves by AMF inoculation, but is subsequently inhibited by salt concentrations (Yano-Melo et al., 2003; Campanelli et al., 2013) and different AMF species (GM, *G. deserticola*, and *G. gergaria*) (Abdel-Fattah and Asrar, 2012). In rice crop cv. Puntal, shoot fresh weight and shoot dry weight of AMF-inoculated plants (*C. etunicatum*) under salt stress (75 and 150 mM NaCl) were greater than those in un-inoculated plants (Porcel et al., 2015, 2016). Likewise, number of panicles, panicle weight, grain yield, and 1000-grain weight in rice crop inoculated with AMF (*Sebacina vermifera*) and subjected to salt stress (3, 6, and 9 dS m^–1^ EC_e_ NaCl) performed better than that of un-inoculated plants, but again depending on the degree of salt stress (Pirdashti et al., 2012). In wheat cvs. Sids 1 and Giza 168, number of grains per plant and grain yield per plant were significantly improved by AMF-inoculation (mixed *Glomus* spp.) under salt stress [4.7 and 9.4 dS m^–1^ (a mixture of NaCl, CaCl_2_ and MgSO_4_ at molar ratio of 2:2:1)] (Talaat and Shawky, 2014). In maize, the salt tolerant abilities (66 and 100 mM NaCl) in terms of shoot dry weight of plants subjected to different AMF strains, *R. intraradices* and *C. etunicatum*, were significantly improved than in plants without AMF and those inoculated with *S. claroideum* (Estrada et al., 2013a, b). Moreover, yield per pot, 1000-grain weight, and grains per ear of AMF-inoculated (*Piriformospora indica*) barley cvs. Ingrid and Annabell grown under salt stress were greater than that of the un-inoculated plants (Waller et al., 2005).

### Anthocyanin Analysis

**FIGURE 7:**
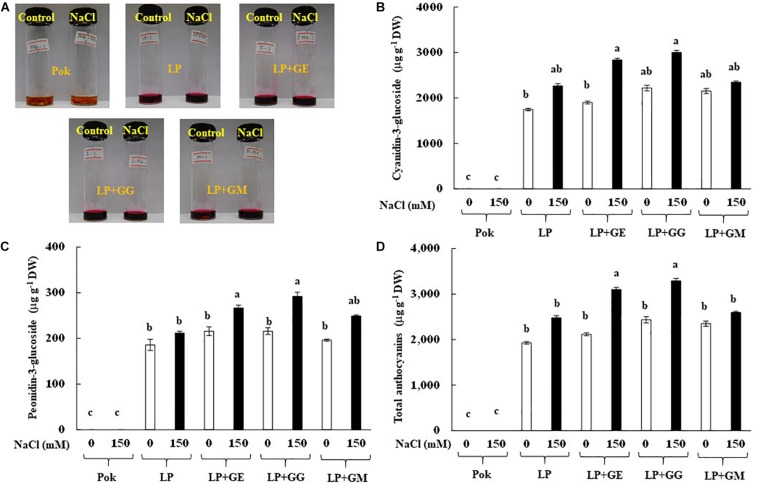
HCl-methanolic extracted solution of rice grain **(A)**, cyanidin-3-glucoside (C3G; **B**), peonidin-3-glucoside (P3G; **C**), and total anthocyanins **(D)** of “Leum Pua” (LP) plants inoculated with AMF (GE; *Glomus etunicatum*, GG; *G. geosporum*, and GM; *G. mosseae*) at booting stage, and subsequently recovered until grain harvesting process. Error bar in each treatment represents by ± SE (*n* = 6). Different letters in each bar represent significant difference at *p* ≤ 0.01 according to Tukey’s HSD.

**FIGURE 8:**
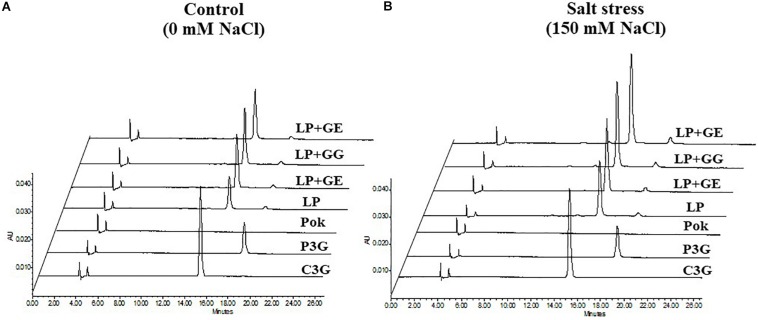
HPLC profiles of cyanidin-3-glucoside (C3G) and peonidin-3-glucoside (P3G) of “Leum Pua” (LP) plants inoculated with AMF (GE; *Glomus etunicatum*, GG; *G. geosporum*, and GM; *G. mosseae*) at booting stage exposed to control **(A)** and salt stress **(B)** conditions for 14 days, and subsequently recovered until grain harvesting process.

Extracted solution of anthocyanins using 1% HCl in methanol solvent is presented in Figure 7A. Interestingly, C3G, P3G, and total anthocyanins in cv. Pok were absent, whereas these were accumulated in the pericarp of cv. LP, especially in the AMF-inoculated plants subjected to salt stress (Figures 7B–D). In LP + GE, C3G, P3G, and total anthocyanins in salt stressed pericarp of rice grains were increased by 1.49, 1.24, and 1.47 folds over the control, respectively (Figures 7B–D). P3G and total anthocyanins in LP + GG under salt stress were significantly increased by 1.35 and 1.35% over the control, respectively (Figures 7C,D). In addition, the regulation of C3G and P3G chromatogram profiles in LP rice cultivar under salt stress was evidently demonstrated (Figure 8).

Total anthocyanin enrichment in the pericarp of rice grain depends on genotype and the biotic and/or abiotic environmental elicitors. In the present study, anthocyanins in the red pericarp of Pok were absent, whereas two species of anthocyanins, C3G and P3G, were present in cv. LP. In lettuce, anthocyanins in the inner and outer leaves of cv. Maravilla de Verano (MV) were accumulated in higher amounts than Batavia RubiaMurguia (BRM) (Baslam et al., 2011). Anthocyanins were evidently dominated in the stem and whole plant of basil varieties, i.e., Cinnamon, Siam Queen, Sweet Dani, and Red Rubin, whereas these were undetected in the roots (Seagel, 2012). In addition, anthocyanin accumulation in pericarp of rice grain inoculated by AMF and exposed to NaCl salt elicitor was clearly observed. In strawberry fruits, C3G, P3G, and pelagonidin-3-rutinoside (P3R) were alleviated in plants grown under AMF (*Glomus* sp.) + *Pseudomonas* bacteria + 70% fertilization (Lingua et al., 2013). Total anthocyanins were increased in lettuce in AMF (commercial inoculation; mixed *G. intraradices* and GM) inoculated plants compared to plants without AMF (Baslam et al., 2011). It is possible that AMF and NaCl salt may regulate the anthocyanin biosynthesis pathway, via targeting several enzymes, i.e., phenylalanine ammonialyase (PAL), chalcone synthase (CHS), and flavonol synthase (FLS) (Abdallah et al., 2016; Battini et al., 2016). In contrast, proanthocyanidins in the leaves of AMF (*Gigaspora albida* and *Acaulospora longula*) inoculated “Aroeira-do-sertão” were unchanged when compared to plants without inoculation (da Silva and Maia, 2018). Likewise, total anthocyanins declined in the leaves of *Cicer arietinum* cvs. PGB5 (salt tolerant) and BG256 (salt susceptible), inoculated with AMF (*R. intraradices*) in response to the degree of NaCl salt treatments (Garg and Bharti, 2018). Moreover, accumulation of anthocyanins in rice grain varies with the species of *Glomus* genus, as seen from the greater accumulation of anthocyanins in grains-derived from GG and GE pretreated plants compared to plants with GM inoculation. In lettuce cvs. Cogollos de Tudela, BRM, and Maravia de Verano, GF, *G. intraradices*, and GM evidently regulated carotenoids (neoxanthin, violaxanthin, antheraxanthin, zeaxanthin, lutein, lactucaxanthin, and β-carotene) and tocopherols (α-, β-, and γ- tocopherols), thereby demonstrating their role as biotic elicitors (Baslam et al., 2013).

## Conclusion

**FIGURE 9:**
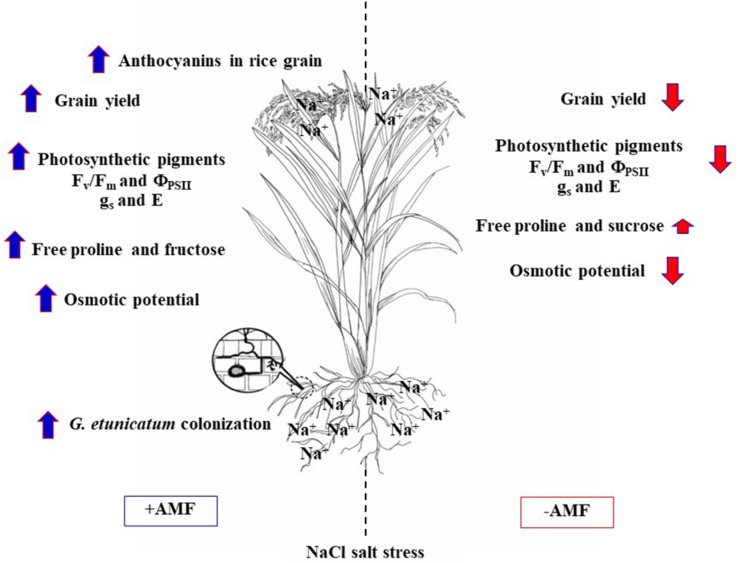
A summary of GE (*Glomus etunicatum*) regulation on salt tolerant ability of “Leum Pua” (LP) plants at booting stage and subsequently recovered until grain harvesting process.

Root colonization by GE, GG, and GM was detected irrespective of the salt treatment. GG inoculation leads to high level of phosphorus accumulation in flag leaf of rice crop cv. LP, whereas Na^+^ was trend to increase in salt-treated plants similar to cv. Pok (salt tolerant). Photosynthetic abilities, chlorophyll pigments, Chl_a_ fluorescence, and stomatal function in flag leaf of LP inoculated with GE grown under salt stress were stabilized by the production of total soluble sugars and free proline that acted as osmolytes to reduce salt toxicity. Therefore, the yield attributes were maintained, and anthocyanins content was enhanced in the pericarp of rice cv. LP inoculated with GE (Figure 9).

## Data Availability Statement

All datasets for this study are included in the article/Supplementary Material.

## Author Contributions

SC carried out the experiment, data analysis, a draft of manuscript preparation, and played a role as corresponding author. RT analyzed anthocyanins (C3G and P3G) and physiological data. CT did soluble sugar analysis and data analysis. TS assayed free proline and overall growth performances. KP conducted the experiment, AMF-colonization and total phosphorus analysis, and yield attributes. PT played a role as project coordinator and a discussion on manuscript preparation. HS performed a critical reading, comments, suggestion, and grammatical checking before submission.

## Conflict of Interest

The authors declare that the research was conducted in the absence of any commercial or financial relationships that could be construed as a potential conflict of interest.
