# Supplementary material for: Alleviation of Salt Stress in Upland Rice (Oryza sativa L. ssp. indica cv. Leum Pua) Using Arbuscular Mycorrhizal Fungi Inoculation
Source: Front Plant Sci. 2020 Mar 26;11:348. doi: 10.3389/fpls.2020.00348 (PMC7113393; doi:10.3389/fpls.2020.00348)
Supplement: Supplementary file 1 [file Presentation_1.PPTX]

## Slide 1
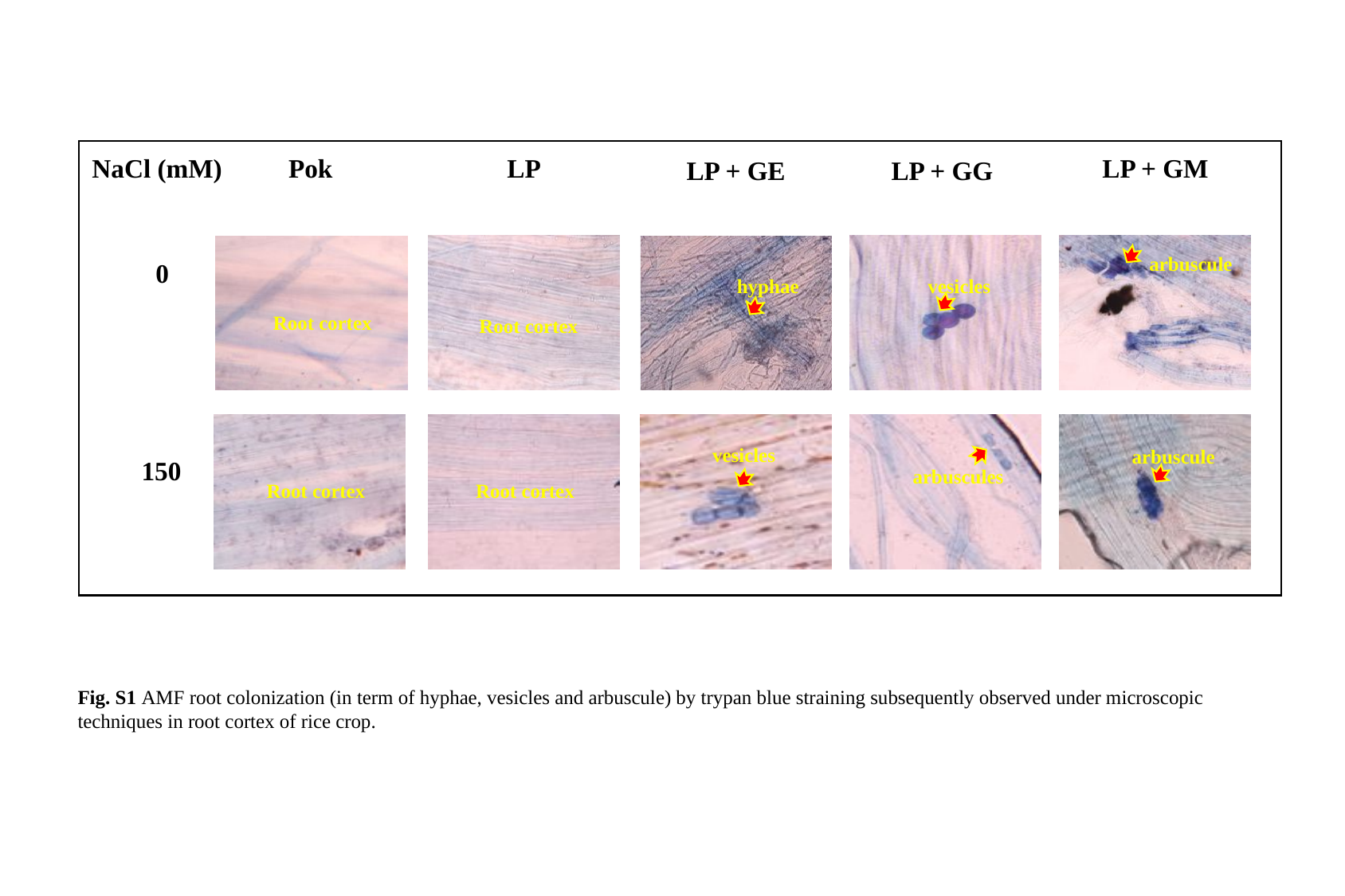

Pok
LP
LP + GM
NaCl (mM)
LP + GE
LP + GG
arbuscule
0
vesicles
hyphae
Root cortex
Root cortex
vesicles
arbuscule
150
arbuscules
Root cortex
Root cortex
Fig. S1 AMF root colonization (in term of hyphae, vesicles and arbuscule) by trypan blue straining subsequently observed under microscopic techniques in root cortex of rice crop.

## Slide 2
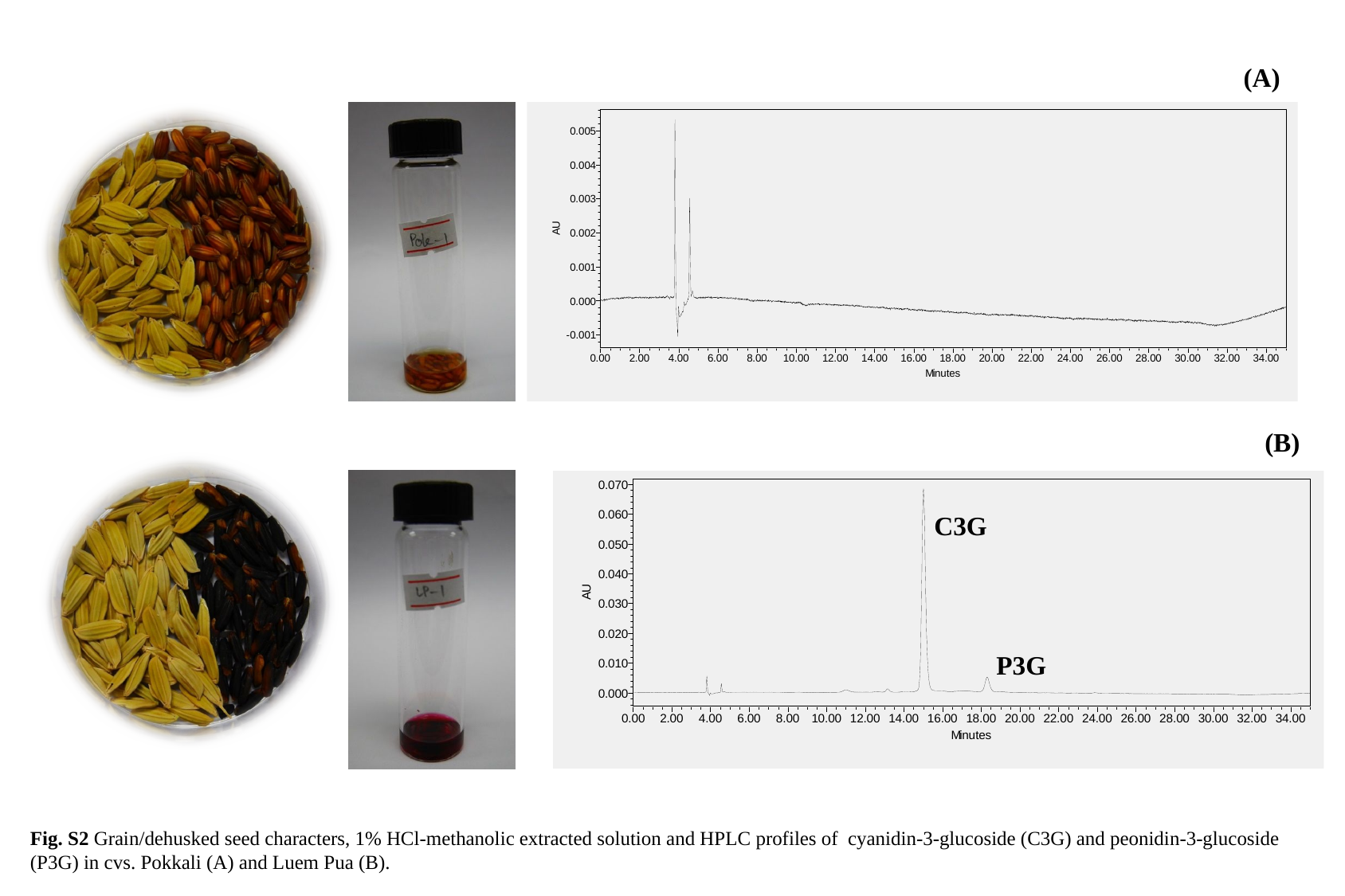

(A)
(B)
C3G
P3G
Fig. S2 Grain/dehusked seed characters, 1% HCl-methanolic extracted solution and HPLC profiles of cyanidin-3-glucoside (C3G) and peonidin-3-glucoside (P3G) in cvs. Pokkali (A) and Luem Pua (B).

## Slide 3
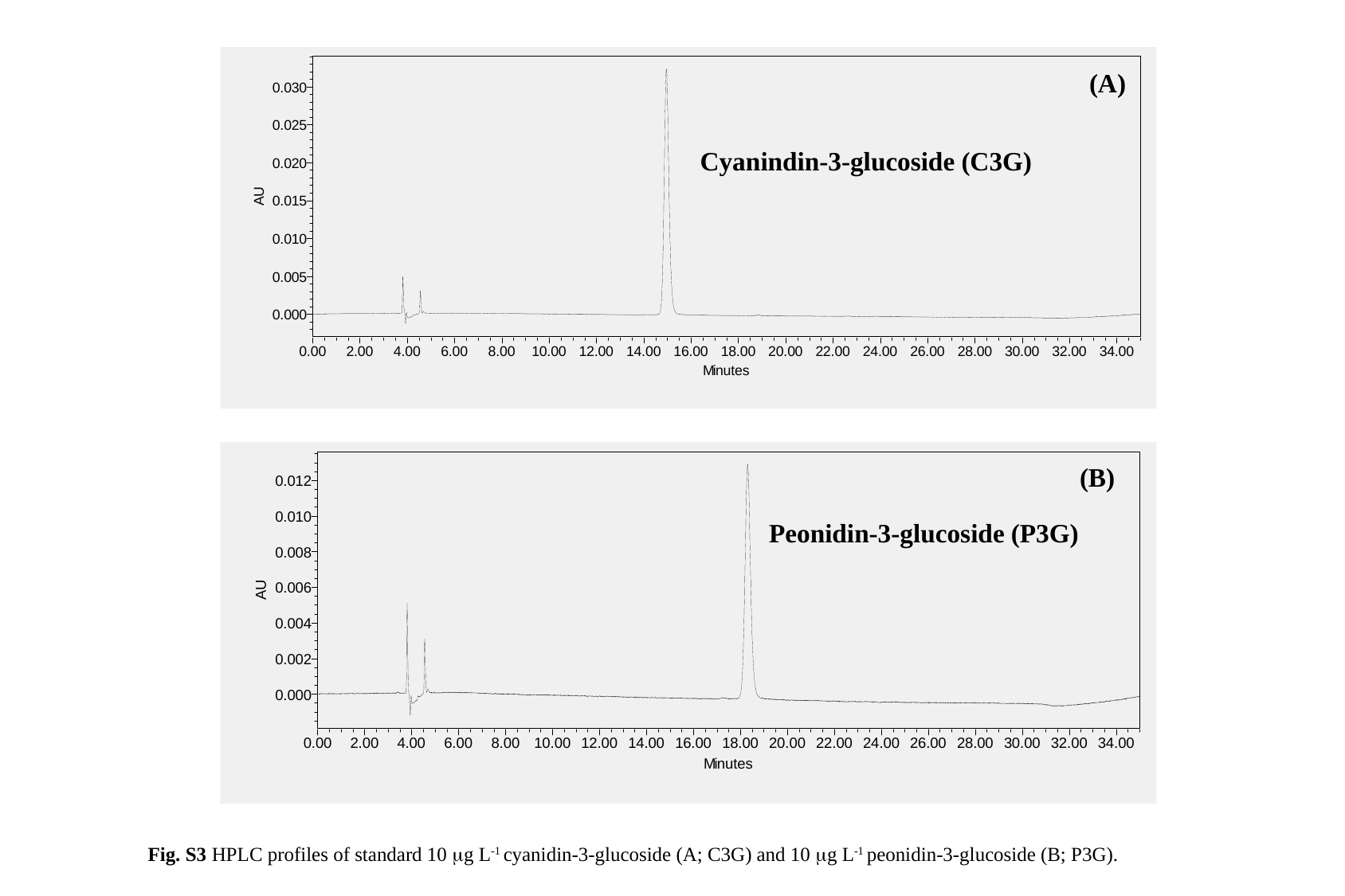

(A)
Cyanindin-3-glucoside (C3G)
(B)
Peonidin-3-glucoside (P3G)
Fig. S3 HPLC profiles of standard 10 mg L-1 cyanidin-3-glucoside (A; C3G) and 10 mg L-1 peonidin-3-glucoside (B; P3G).

## Slide 4
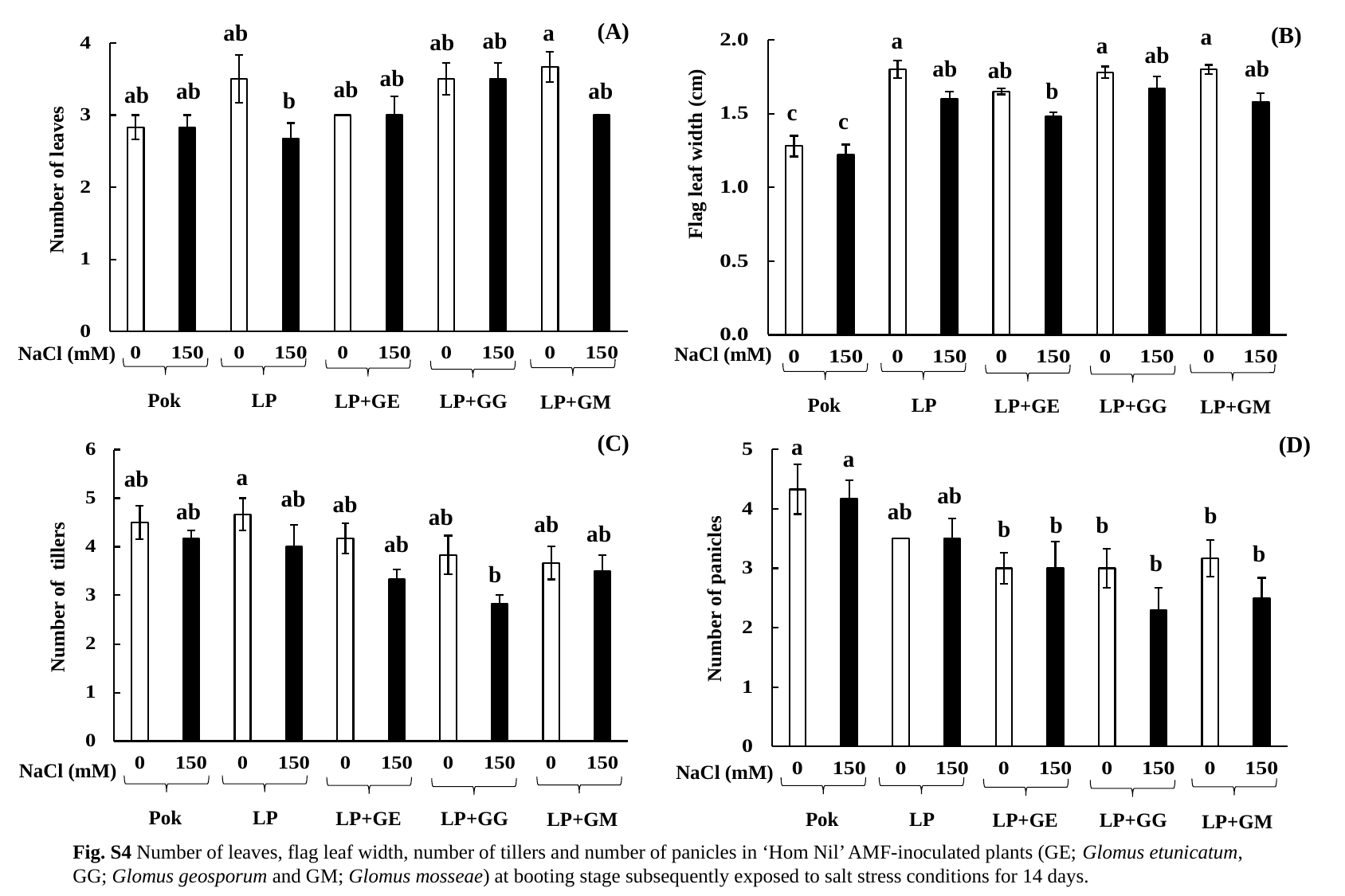

(A)
ab
a
(B)
a
a
ab
ab
a
ab
ab
ab
ab
ab
Flag leaf width (cm)
ab
ab
ab
b
ab
b
c
Number of leaves
c
NaCl (mM)
NaCl (mM)
Pok
LP
LP+GE
LP+GG
LP+GM
Pok
LP
LP+GE
LP+GG
LP+GM
(C)
(D)
a
a
a
ab
ab
ab
ab
ab
ab
b
ab
ab
b
b
b
Number of panicles
ab
Number of tillers
ab
b
b
b
NaCl (mM)
NaCl (mM)
Pok
LP
LP+GE
LP+GG
LP+GM
Pok
LP
LP+GE
LP+GG
LP+GM
Fig. S4 Number of leaves, flag leaf width, number of tillers and number of panicles in ‘Hom Nil’ AMF-inoculated plants (GE; Glomus etunicatum, GG; Glomus geosporum and GM; Glomus mosseae) at booting stage subsequently exposed to salt stress conditions for 14 days.
